# Recognition of Pashto Handwritten Characters Based on Deep Learning

**DOI:** 10.3390/s20205884

**Published:** 2020-10-17

**Authors:** Muhammad Sadiq Amin, Siddiqui Muhammad Yasir, Hyunsik Ahn

**Affiliations:** Department of Robot System Engineering, Tongmyong University, Busan 48520, Korea; msamin@tu.ac.kr (M.S.A.); mysi@tu.ac.kr (S.M.Y.)

**Keywords:** deep learning, deep features fusion, convolutional neural networks, computer vision, Pashto handwritten character recognition

## Abstract

Handwritten character recognition is increasingly important in a variety of automation fields, for example, authentication of bank signatures, identification of ZIP codes on letter addresses, and forensic evidence. Despite improved object recognition technologies, Pashto’s hand-written character recognition (PHCR) remains largely unsolved due to the presence of many enigmatic hand-written characters, enormously cursive Pashto characters, and lack of research attention. We propose a convolutional neural network (CNN) model for recognition of Pashto hand-written characters for the first time in an unrestricted environment. Firstly, a novel Pashto handwritten character data set, “Poha”, for 44 characters is constructed. For preprocessing, deep fusion image processing techniques and noise reduction for text optimization are applied. A CNN model optimized in the number of convolutional layers and their parameters outperformed common deep models in terms of accuracy. Moreover, a set of benchmark popular CNN models applied to Poha is evaluated and compared with the proposed model. The obtained experimental results show that the proposed model is superior to other models with test accuracy of 99.64 percent for PHCR. The results indicate that our model may be a strong candidate for handwritten character recognition and automated PHCR applications.

## 1. Introduction

Handwritten character recognition is considered to be one of the most challenging and appealing research areas in the field of pattern recognition and computer vision. Due to the critical factors of differences in writing patterns and cursive text, and the similarity of various characters in the form, recognition research is time-consuming and challenging. Recognition of handwritten characters may be performed online or offline. Online character identification is relatively simple due to the temporal-based character properties such as form, number of strokes, distance, and direction of writing. Offline character recognition implementation is complex due to variations of writers and fonts. The literature shows a high accuracy rate for recognition of characters and isolated words in optical character recognition (OCR) or printed text; however, there is a need for a competent handwritten character recognition system capable of generating a high degree of accuracy in handwritten text recognition [1,2,3,4].

Pashto is Afghanistan’s official language and Pakistan’s second-most spoken and written language [5]. Pashto script is written from right to left, and is known as a bidirectional language, and includes Arabic, Persian, and Urdu languages. All Pashto-script-based languages have special properties. For example, these languages have cursive letters drawn from right to left, in contrast to English or other Western letters. In addition, the script of these languages depends on the context, i.e., the script of certain languages incorporates more than one character to convey words. The words are written in ligature form by characters; as a result, every character has a different shape in different words depending on its positioning in the word.

Based on a review of the literature, it is apparent that inadequate work exists on the recognition of Pashto handwritten characters compared to other foreign language scripts [6,7,8]. Various machine learning techniques, such as ANNs (artificial neural networks) [9] and KNN (K-Nearest Neighbors) [10], have been applied to Pashto handwritten character recognition [11]. However, the accuracy of the results was not sufficient for real character recognition applications.

By comparison, convolutional neural networks [12,13,14] have been demonstrated to be effective in pattern recognition for images and have been commonly used in computer vision tasks. The strength of deep neural networks is that they offer excellent performance without taking into account whether the underlying essence of the deep model is linear.

The advantage of a convolutional neural networks (CNN) advantage is its applicability to character recognition, such as OCR and hand-written character recognition (HCR), for nearly any available language, i.e., English, Arabic, Hangul, etc. However, the research related to Pashto handwritten character recognition (PHCR) lacks application of deep learning models, particularly CNN and other popular deep neural network models. The current paper fills this research gap by suggesting an optimal model that is easy to train and yields the best test accuracy, outperforming benchmark deep neural network models.

In this paper, Pashto handwritten character recognition using an optimal CNN model and a large-scale data set is proposed. Furthermore, we construct a public Pashto handwritten character data set (Poha) written by native Pashto speakers and foreigners.

The contributions of this study are as follows. First, a convolutional neural network was developed for Pashto HCR that achieves optimum accuracy. Second, a new data set of Pashto HCR was constructed and made publicly available for further research in this domain. The third contribution is a comparison of the developed model, using the Poha dataset, to benchmark deep neural network models; results showed that the proposed model achieved high accuracy. In addition, the proposed model can be effective in automated educational applications for Pashto character learning and writing. The model can intelligently classify Pashto characters written by children.

The paper is structured as follows: Section 2 provides comprehensive literature review for Pashto and its sister languages. The proposed model is outlined in Section 3 and the experimental results are presented in Section 4. Finally, future research directions and conclusions are noted in Section 5.

## 2. Literature Review

In this section, related work on handwritten character recognition of Arabic, Urdu, and Pashto handwritten characters is described. In handwritten character recognition, features have generally been extracted from input image data. Then, for estimation of posterior probabilities [3,13,15,16,17], classifiers such as Gaussian Mixture (GMM) or artificial neural networks (ANN) and its variants have been widely used. These probabilities are input to the Hidden Markov Model (HMM) [18] to produce transcription. HMMs have drawbacks because they are unsuccessful in modeling long-term dependencies in inputs. However, Recurrent neural network(RNNs) can solve this drawback [19]. RNNs such as Long short-term memory (LSTM) are remarkably good at sequence learning tasks [20].

Many studies have been conducted on handwritten Arabic and Urdu character recognition; however, Pashto character recognition has not been explored to the same extent. Arabic handwritten character recognition can be classified into two approaches, conventional approaches and deep learning-based approaches.

Conventional approaches depend on manual feature extraction by experts whereas deep learning-based techniques automatically extract features from raw images. Conventional techniques cannot extract features from images in their raw form. Machine learning experts have struggled to design feature extractors that extract discriminative features from raw data into vectors as an input to classifiers for pattern recognition [21].

In a notable early work for isolated Arabic character recognition, Abandat et al. [22] suggested principle component analysis. In this study, the authors extracted a subset of 40 features from 95 features. Classifiers including Quadratic Discriminant Analysis (QDA), Linear Discriminant Analysis (LDA), Diagonal QDA (DQDA), Diagonal LDA (DLDA), and KNN (K-Nearest Neighbors) were used for classification. The study achieved 87% accuracy during experimentation with the suggested dataset that contained 4992 characters. Aljuaid et al. [23] proposed a genetic approach for the classification of Arabic handwritten characters. The authors worked on the shapes of Arabic handwritten characters by extracting the features which were related to structural differences between characters. The results of the study achieved accuracy of 87%. Al-jawafi [24] presented a neural network with three layers. This layered approach caused more complexity in computation. The study dataset consisted of 750 segmented characters for both network training and testing, which was not sufficient to improve accuracy using an artificial neural network (ANN). They reported a 0.42 mean square error (mqe on the test dataset.

In the case of deep learning approaches, the use of convolutional neural networks for the recognition of Modified national institute of standards and technology(MNIST) and Extended modified national institute of standards and technology (EMNIST) datasets has attracted the attention of numerous researchers, who have applied these models to languages with handwritten character recognition, including English [25], Hangul [26], and Chinese [27]. Arabic-related research studies using deep learning models and techniques have been reported in this research [28]. The authors first designed an image processing module, and constructed a data set, for mobile devices. They proposed a lightweight CNN for optical character recognition for the dataset. Another work addresses the variability in writer’s handwriting [29], in which a feature-ranking technique was adopted. The authors considered different univariate measures to produce a feature ranking and proposed a greedy search approach for choosing the feature subset able to maximize the classification results. Raymond et al. [30] presented a fully convolutional network architecture that outputs arbitrary length symbol streams from handwritten text. A preprocessing step normalizes input blocks to a canonical representation, which negates the need for costly recurrent symbol alignment correction. The authors introduced a probabilistic character error rate to correct errant word blocks.

A recent study by Chaouki Boufenar et al. [31], investigated the use of convolutional neural networks for offline Arabic handwritten character recognition. Their architecture consisted of five layers in which three convolutional layers with a max pool were connected to two fully connected layers. They used OIHACDB-28 for training and evaluation of the model, gained a result of 97.32% accuracy. The CNN model was trained with a dropout technique under the Theano framework. Ahmed El-Sawy et al. [32] suggested the Deep convolutional neural networks (DCNN)model for the recognition of isolated handwritten Arabic characters. They proposed a dataset referred to as Arabic handwritten characters dataset AHCD. The model was trained with an optimization method for 30 epochs that resulted in a significant increase in performance and a 94.9% classification accuracy.

For the classification and recognition of Urdu characters, the work reported in [33] used Support vector machine (SVM). The experiments were conducted on a dataset that contained 36,800 handwritten characters. The author achieved accuracy of 93.5% for offline Urdu handwritten character recognition. Another study [34] used SVM and the radial base function (RBF). The authors experimented on a dataset that contained 47,151 training set images and 13,178 testing set images, and achieved performance of approximately 98.6%. The authors in this study [35], categorized the number of strokes into four classes. The study proposed online intelligent Urdu character recognition by considering a single stroke character. Three different classifiers, namely, a correlation-based classifier, backpropagation neural network (BPNN), and probabilistic neural network (PNN), were fed with statistical features that were pre-extracted. The dataset contained 85 instances from 35 writers. The author claimed that the PNN achieved the best accuracy of 94%. PNN-based classifiers do not require pre-training, thus resulting in higher accuracy compared to the BNN-based classifier. A comprehensive summary related to Urdu hadnwritten character recognition is provided in Table 1.

Pashto handwritten character recognition has been investigated less than that for Urdu and Arabic. The available literature shows unsatisfactory research results compared to other languages. In [41], the Byblos Pashto OCR system was proposed for script-free OCR using HMMs. This system was also subsequently tested for Chinese, English, and Arabic text with success. As previously mentioned, Pashto Intelligent character recognition(ICR) and Optical character recognition (OCR) area are the least explored to date. Thus, due to the unavailability of a Pashto corpus, the authors of this paper collected 27,000 characters from faxed printed pages. They then scanned these pages using a 300 dpi scanner. This research work lacked in performance due to insufficient training data. In [42], a novel algorithm was proposed, in which complicated inputs are preprocessed and the shape of the actual input is maintained. The secondary stroke is linked to the primary stroke by fuzzy association rules. Numerous classifiers are used, such as fuzzy logic, CNN, KNN, and hybrid fuzzy HMM. The results of the mentioned classifiers were evaluated by statistical tests. In a notable study [43], the authors suggested a Pashto OCR (optical character recognition) system. A small dataset was compiled with 1125 entries. In the proposed approach, individual Pashto characters were recognized by utilizing both high- and low-level features. High-level features were based on the structural information from the characters and the resulting binary trees uniquely classified each of the characters. Although the approach was robust, it was affected by the variation in size, orientation, and writing style. An alternative low-level feature approach based on K-Nearest Neighbors was used giving an overall word recognition of 74.8% [43]. A recent study [44] was conducted on Pashto handwritten numerals (PHNR) based on deep learning. The authors trained CNN and RNN models for feature extraction and classification. They evaluated the results on a newly constructed Pashtu handwritten numerals database (PHND) and Bangla handwritten numerals dataset CMATERDB 3.1.1. The study reported recognition rates of 98.00% for PHND and 98.64% for CMATERDB 3.1.1 datasets.

Based on this discussion, it can be concluded that there is a lack of a Pashto handwritten character dataset. In addition, a research gap exists for classification of Pashto handwritten characters based on deep learning techniques, such as the CNN. The current article fills this gap by proposing a CNN model and a Pashto handwritten character dataset. For this purpose, a model was developed and extensive experimentation conducted. These are described in detail in Section 3 and Section 4.

## 3. Proposed Model

**Figure 1:**
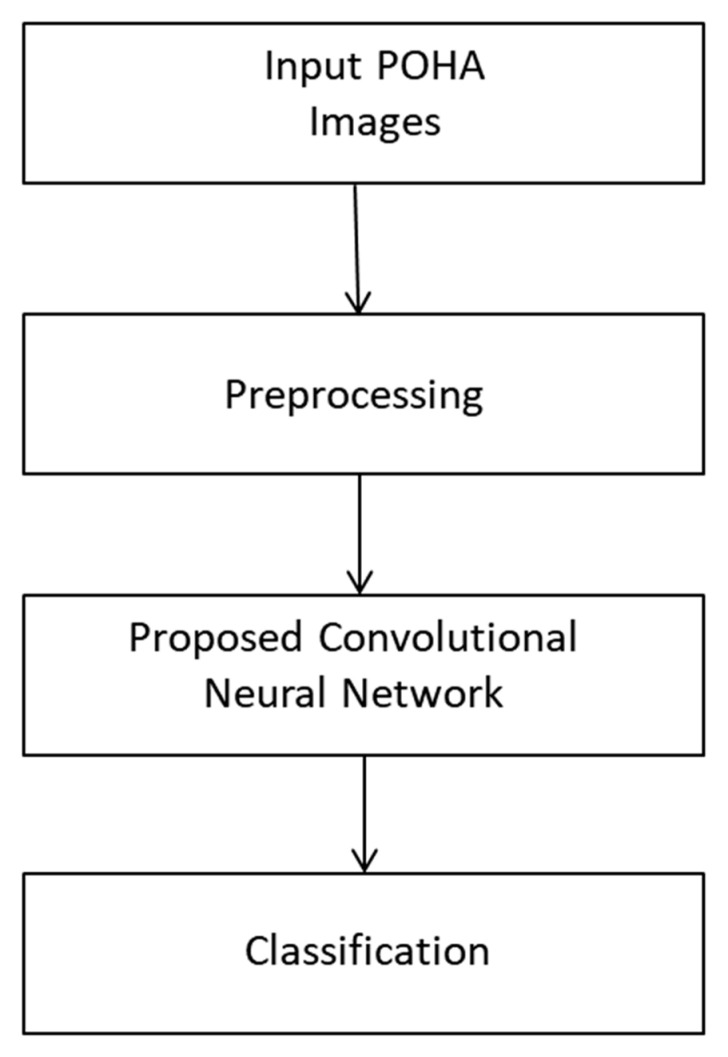
Block diagram of our proposed methodology.

We proposed a means of Pashto handwritten character classification and recognition as shown in Figure 1. Our recognition system relies on a CNN applied for the first time to a Pashto handwritten character dataset with a feature mapped output layer. Our suggested CNN model classifies Pashto characters into 13 classes that contain 44 subclasses. A detailed explanation of the suggested model is presented in the following subsections.

### 3.1. Poha Dataset

**Figure 2:**
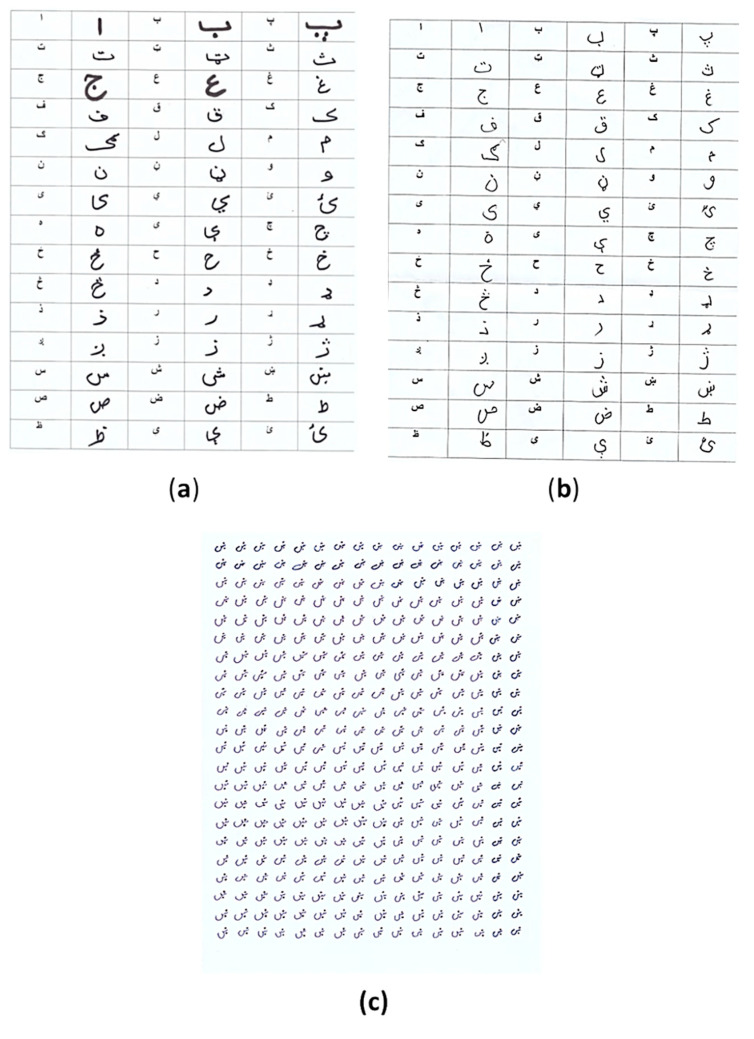
Form distributed to authors and used to compile the dataset: (**a**) The form completed by native Pashto speakers in Khyber Pukhtunkhwa, KPK Pakistan; (**b**) this form completed by non-native speakers in South Korea; (**c**) the individual letter “Khin” written by one user.

A data-enriched dataset plays a vital role in the generation of accurate results in research activities related to deep learning. A concise and precise dataset is required for a true evaluation of mathematical models that are applied to it. Moreover, to achieve benchmark results in deep learning, a standard publicly available dataset is mandatory. During the experimental phase of this research, it was found that no public Pashto handwritten character dataset is available due to the limited research work regarding this language, as mentioned in Section 2. We constructed a new dataset for Pashto handwritten isolated characters to fill this gap for the research community. We named this dataset Poha (Learning). Our dataset contains 26,400 images for each of 44 Pashto characters and 10 numerals. The dataset was constructed by 300 native Pashto speakers from the Department of Pashto, University of Peshawar, Kyber Pukhtunkwa (KPK), Pakistan. These Pashto speakers were split into three groups. The first party consisted of six faculty members, most of whom had Ph.D. degrees in Pashto literature. The second group consisted of 20 master students, and the remainder were undergrads. Each author was instructed to write a Pashto character on the page we distributed, shown in Figure 2.

Our main priority was to gain a high degree of accuracy for character recognition. Hence, non-native Pashto speakers from Tongmyoung University Busan, South Korea, who are Korean students and faculty members, were also invited to contribute to the study. A total of 50 students and 3 faculty members participated in writing Pashto isolated characters and numerals. We provided the same page as that given to the native Pashto speakers. We observed that the native speakers were more confident in writing than the non-native speakers. The writing style of non-native speakers was different from that of native speakers; for example, Koreans write from left to right whereas native speakers write from right to left. Involving two different groups addresses the issue of model overfitting due to diverse handwriting style samples. We recorded the detailed information of the writers who participated in the construction of our proposed dataset in a separate database, which is available online on our lab website. These details include age, name, gender, handwriting preferences, physical difficulties, and occupation. We scanned text pages using a flatbed HP scanner at 600 dpi following the compilation of the dataset. The size of the images was converted to 28 × 28 for each Pashto character. The number of characters in our proposed dataset is 600 × 44 = 26,400.

### 3.2. Preprocessing

Preprocessing steps were applied to the proposed dataset to prepare images for subsequent phases. Image preprocessing consisted of operations on images at the lower abstraction level with the aim of improving the image data. This improvement suppresses undesired distortions in the image dataset, or enhances important details or features that are essential for further processing.

**Figure 3:**
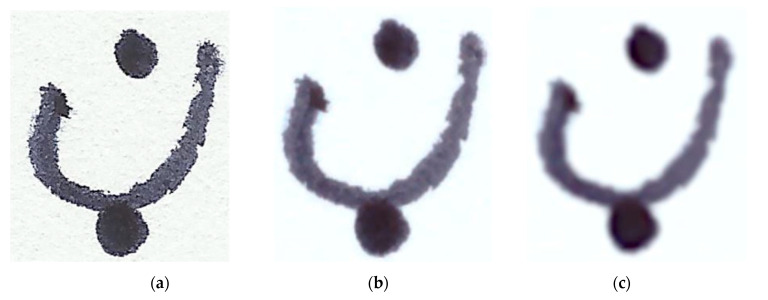
Image dataset denoising steps: (**a**) scanner-induced noise; (**b**) denoised image; (**c**) application of Gaussian blur filter.

The first step of the preprocessing phase was the removal of noise from images using Gaussian blur. The digital scanner induced spikes of noise into the scanned images, as shown in Figure 3a. These spikes of noise were removed using the notable work of Soman [45]. The second step was smoothing of the image using the Gaussian function. This can be considered to be low-pass filtering in a non-uniform manner, which conserves the low frequency, and decreases the noise and insignificant details in an image. This was accomplished by convolving the Gaussian kernel with an image.

(1) $$G_{2D}\left(x,\;y,\;\sigma \right)\;=\;\frac{1}{2\pi \sigma^{2}}e^{-}\frac{x^{2}+y^{2}}{2\sigma^{2}}$$

where σ is the standard deviation, and x and y are location indices. The standard deviation controls the variance around the Gaussian distribution mean value, and establishes the blurring effect around certain pixels. In our study, we used σ = 3, which generates a good smoothing effect to suppress the scanner-induced noise. The images were then converted to gray-scale and, finally, the images were resized to 28 × 28 pixels and the aspect ratio was held constant. A CNN was subsequently used for detection and classification of features.

### 3.3. Convolutional Neural Network

The architecture of the CNN differs from that of typical neural networks. The CNN is designed to emulate the visual processing system of humans, and has distinctly enhanced structures for 2D image processing. The capabilities of the CNN to grasp the abstraction and extraction of 2D features are effectual. In addition, CNN’s max-pooling layer shows its efficacy by identifying shape variations. The weights are tied to a sparse network of the CNN that requires fewer parameters than fully connected networks of similar size. Furthermore, the CNN is trainable using a gradient-based learning algorithm that is effective in overcoming the decreasing gradient problem. Thus, the gradient-based algorithm trains the entire network to diminish the error criterion, which results in better generalization and optimized weights.

The general architecture of the CNN consists of two main units: classifier and feature extractor. Each layer of the network inside the feature extraction unit receives input from the output of the previous layer and feeds the current output into the next layer as an input. The classifier unit predicts the output data with corresponding input data. As can be seen in Table 2, there are two basic layers in feature extraction: convolutional and pooling layers [46].

In convolutional layers, each node applies convolutional operations on input nodes to extract the features from input image data. The input of the *nth* layer is the output of the n−1th layer, whereas this input passes through a set of filters or kernels followed by nonlinear activation of rectified linear unit (ReLU) functions. For example, if f represent the activation function of ReLU, $x_{j}^{n-1}$ is an input from the n−1  layer, $k_{i,j}^{n}$ are the filters of the *n*th layer, and biases of the *n*th layer are represented as $b_{i,j}^{n}$, then the convolution layer operates as:

(2) $$x_{j}^{n}\;=\;f\left(x_{i}^{n-1}*\;k_{i,j}^{n}\right)+b_{i,j}^{n}$$

The pooling layer applies maximum or averaging operations on the input nodes that abstract features. For instance, if a 2 × 2 downsampling filter or kernel is applied to the input of the pooling layer, the output dimension will be reduced to one-half of the related input dimension for all inputs.

The operation of pooling can be expressed as follows:

(3) $$\;\;\;x_{j}^{n}\;=\;\mathrm{down}\;x_{i}^{n-1}$$

Conventional neural networks extract high- to low-level features; however, the CNN extracts features from low to high level, in contrast to neural networks. These higher-level features are obtained from the propagation of inputs from the lower level. This propagation reduces the dimension of features depending on the mask size of pooling and convolution. However, the amount of feature mapping escalates for the selection of optimized features of input images for higher classification accuracy. As shown in Table 2, the output of the last layer is used as an input to the fully connected layers that use the Softmax operation to classify inputs. For example, for a weight vector *w*, a sample input *x*, and linear function *K*, for the *i*th class, the Softmax operation is denoted mathematically as:

(4) $$P\left(y=i|x\right)\;=\;\frac{\exp\left(x^{T}w^{i}\right)}{\sum_{K=1}^{K}\exp\left(x^{T}w^{K}\right)}$$

In conventional neural networks, each layer consists of a set of neurons. Input to these networks is transfigured across a set of hidden layers that are interconnected by neurons to previous and following layers. The performance of the CNN is higher than that of conventional neural networks due to the traversing immanent characteristics of images [47]. This prominent and improved performance feature of the CNN motivated us to use it on the proposed Poha dataset.

The CNN model was fed with input images from our Poha dataset with a size of 28 × 28 pixels per image. The first layer of our model was the 2D convolutional layer with a kernel size of 3 × 3. This layer uses every pixel of the input image. The outcome of this layer was implanted with a 26 × 26 feature map. This feature map was embedded with the geometrical features to set up a feature vector. A ReLU (rectified linear unit) [48] was used as an activation function to activate every output of the convolutional layer. ReLU was used rather than the “Sigmoid” function [49] because of its capability to address the problem of gradient vanishing. A ReLU uses the inherent threshold invariant for stimulation that is similar to the human brain mechanism. The input of the first convolutional layer was fed into a max pool for nonlinear downsampling with a stride of 2. The output of this layer was 13 × 13 feature vectors which were implanted into the second convolutional layer. A mask of the kernel with a size of 3 × 3 was applied to the input. The output of this layer was again fed to a max pool layer for nonlinear downsampling with a stride of 2. This layer generated an output of size 5 × 5 that was fed into the third 2D convolutional layer. The output of these layers was passed to flattening for a 1D feature vector. This flattened layer was needed to make use of a fully connected layer after the convolutional and max-pooling layers. At the end of our model, the two fully connected (Dense) layers, which are artificial neural networks (ANNs), are used. The last fully-connected layer (Dense (44, activation = ”Softmax”)) classifies output using the distribution of probabilities of each class. The Adam optimizer is used for adaptive learning optimization. Adam can be seen as the combination of the RMSprop and Stochastic Gradient Descent algorithm. The Adam optimizer was selected for our model due to its use of square gradients to scale the learning rate, as used in RMSprop; in addition, Adam takes advantage of momentum by moving the average of the gradient rather than the gradient itself, which is similar to Stochastic gradient descent SGD with momentum [50]. The default setting of the optimizer was change during this implementation to generate optimal results.

During the experimentation phase, different layers and, learning rates, momentum, optimizers, and datasets of size 28 × 28 were tested. However, the optimal results were generated with the model as described in Table 2. The model performed better with fewer parameters, and the computation and resource consumption were lower than that of other models.

## 4. Experimental Setup and Results

The experiment was conducted on a desktop computer with an Intel ^®^ Xeon octa-core 3.3 GHz central processing unit (CPU), 16 GB of memory capacity, and a GeForce GTX 1080Ti graphics card mounted on board. Deep learning libraries, such as Cudnn and Karas with Tensorflow-GPU version 2.20 with the Ubuntu 16.04 operating system were installed on the system. Python was chosen as the programming language for constructing the CNN because it is widely recognized in the research community as a powerful programming language. In addition, image processing libraries specifically designed for Python, such as OpenCV, Numpy, Pandas, and Scipy, were used during model implementation and comparisons.

Several experiments tested the efficiency of the proposed model for classifying handwritten characters. For classification, the Poha dataset was split into two portions: three-quarters of the 26,400 images were used as a training dataset, and the remaining one-quarter were used as a testing set, for 44 classes. During the training process, the iterations, accuracy of training, learning rate, and hidden neurons were considered to be parameters for our proposed convolutional neural network. During experimentation, an improvement in performance was observed, but with the downside of overfitting due to training the model for a longer time. A possible solution for the model overfitting problem is batch size adjustment. A standard training concept is the idea that a model cannot be trained when the batch size is exceeded by a certain threshold [51]. Moreover, batch size relies on available machine memory size. To avoid the problems discussed above, we trained the model using a momentum value of 0.8, holding the batch size equal to 32 and setting the learning rate to 0.0015 in a controlled environment. These particular values showed their significance for optimized results. Search for the best network state and avoiding fitting issues would have progressively expanded the convolutional cores, despite an all-in-one increase. Furthermore, the batch size is required to be large enough to attain the global gradient.

**Figure 4:**
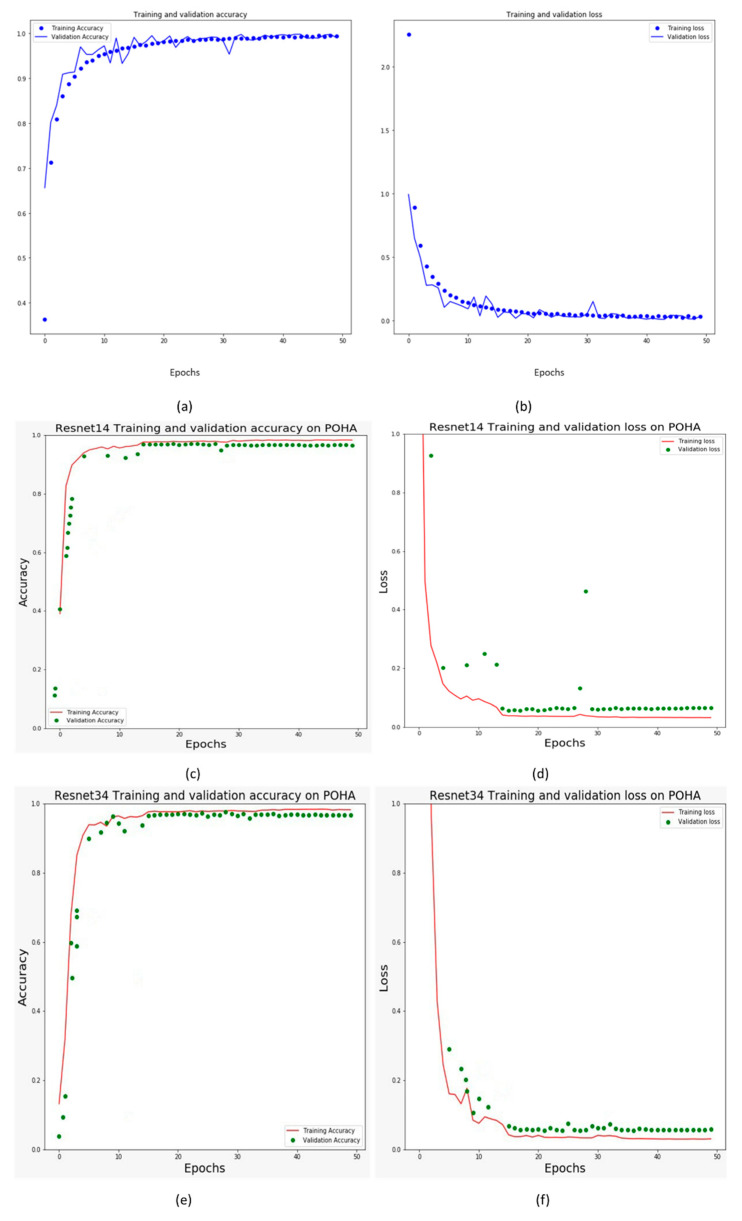
Graph depicting the accuracy of the proposed model, ResNet18, and Resnet34 on the Poha dataset: (**a**) training and validation accuracy; (**b**) training and validation loss; (**c**) ResNet 18 training and validation accuracy; (**d**) ResNet 18 training and validation loss; (**e**) ResNet 34 training and validation accuracy; (**f**) ResNet 34 training and validation loss.

**Figure A1:**
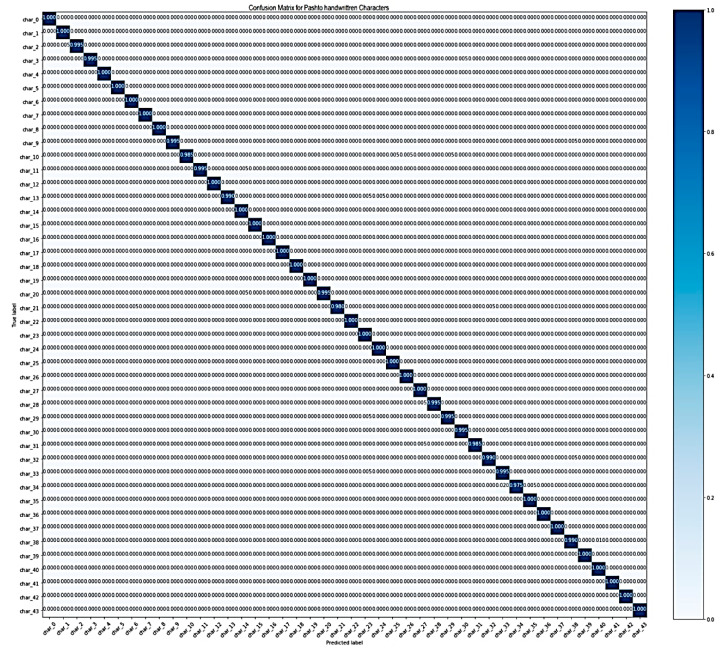
A confusion matrix for 44 classes of Pashto handwritten characters.

For Pashto handwritten characters, the confusion matrix, which shows an attained average accuracy of 99.64%, is shown in Figure A1 in the Appendix A. A 5k-fold validation approach was adopted for cross-validation. In the confusion matrix, diagonal values indicate the classification accuracy of particular Pashto handwritten characters, whereas the non-highlighted boxes in the related confusion matrix show the general achieved accuracy in each experiment. Moreover, the diagonal boxes that are not highlighted relate to incorrect classifications. Each cell contains the number of observations. In the confusion matrix, the right column contains the percentage value of the correct and incorrect prediction value of every class. The performance graph indicates the rise in the iterations with the increase in the number of hidden neurons that achieves optimal accuracy. The number of neurons should not be misinterpreted as the number of classes. In general, the number of hidden neurons is equal to the number of interactions that must be made in each hidden layer. Internal layers of a CNN can be composed of various hidden neurons that help to more deeply choose different features in an input image. It is important to note that the increase in hidden neurons increase the complexity of the network but also supports optimal accuracy. The output shows that the labels of Pashto handwritten characters range from 0 to 43 from a total of 44 classes. A series of experiments was performed for Pashto handwritten characters. We achieved 99.64% accuracy for Pashto handwritten characters as showen in Figure 4, which is a higher degree of accuracy than that of the literature relating to Pashto handwritten characters available to date. Table A1 in the Appendix A describes the classification matrices, namely, F1 score, recall, and precision, for each character class of the Poha dataset.

We trained ResNet 18 [52] and ResNet 34 without any pre-trained weights on the Poha dataset. These models have been designed for general classification tasks such as shape or object classification. However, we decided to conduct experiments with a character dataset for comparison with our proposed model as a research problem. The Poha dataset was carefully sliced into 224 x 224 as an input to these networks. The other parameters, such as batch size, epochs, and learning rate, were maintained at 32, 50, and 0.001, respectively. Figure 4 show the validation accuracy and validation loss for these models trained on Poha. The evaluation parameters, namely, sensitivity, precision, and F1 score, were calculated from these experiments, as shown in Table 3. ResNet 34 exhibited a test accuracy of 97.48% with a test loss of 0.05072. ResNet 18 achieved an accuracy of 98.21% and a test loss of 0.0314 when trained on the Poha dataset without any pre-trained weights. It should be noted that the 5k-fold validation approach was applied during the training process. The experimental comparison results showed that our model performed better than other well-known networks when using the same experimental parameters. We trained well-known models on the Poha dataset without any pre-trained weights (which are usually Imagenet weights). In general, deep, complex CNN models are constructed for complex problems. They are more generalized models for general problems, such as the shape of objects. The experiments show that the performance of deep models is still better for character recognition, however, the performance slightly lags that of the proposed model.

The proposed approach was also compared with the Pashto handwritten character recognition approaches described in Table 4. These approaches are based on features such as zoning, geometrical, and statistical geometry The use of the proposed CNN resulted in optimal performance compared to KNN and ANN, which have previously been used for Pashto character recognition. Because there is limited previous research available for Pashto handwritten characters for comparison, Table 4 compares our approach with other previous machine learning ML approaches related to Pashto handwritten character recognition. The results show that CNN performs better than the mentioned approaches.

**Figure 5:**
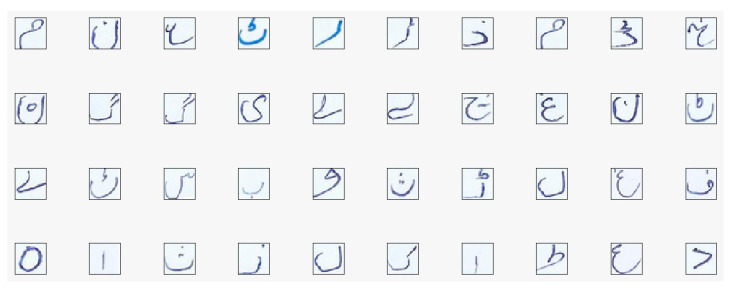
A sample of input images for the proposed model from the Urdu dataset [42].

During experimentation, the dataset of [42] was fed to our model. This dataset contains 40 Urdu characters with 500 images for each character, that is, 500 × 40 = 20,000 character images. The input size of the dataset was 28 × 28. Our proposed model achieved an improved accuracy of 99%, which is 2.96% better than the implementation in [42]. The authors of this paper used geometrical feature extraction of each character in their dataset in a vector before feeding it into the CNN. It was found during experimentation that this technique does not impact the accuracy of recognition and classification of characters. CNN models extract various features by convolutional layers from images, thus feeding a separate feature vector is absurd. Figure 5 shows an input from the Urdu dataset that was fed to our proposed CNN model. The model was also evaluated using another public Devanagari Handwritten Character Dataset [54] containing 36 characters. Each class contains 2000 images per character. The dataset was divided into 1700 training images and 300 test images during experimentation. The size of the input images was 28 × 28. The learning rates, number of epochs, and batch size were 0.0015, 50, and 32, respectively, for both experiments. It should be noted that throughout the experimentation the 5k-fold validation approach was applied to generate the validation accuracy. The results of these experiments on the proposed model are shown in Table 5.

**Figure A2:**
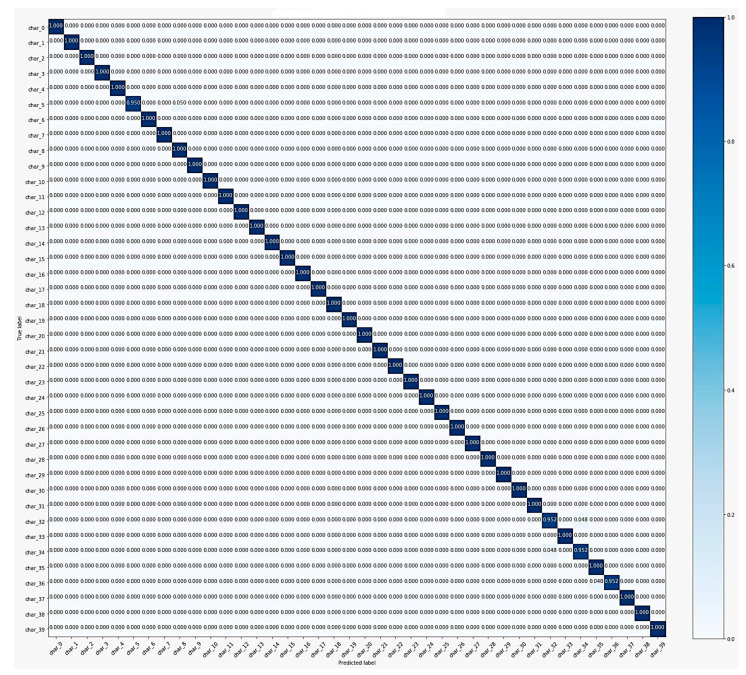
Confusion matrix for the Urdu dataset that contains 40 classes of characters [42].

**Figure A3:**
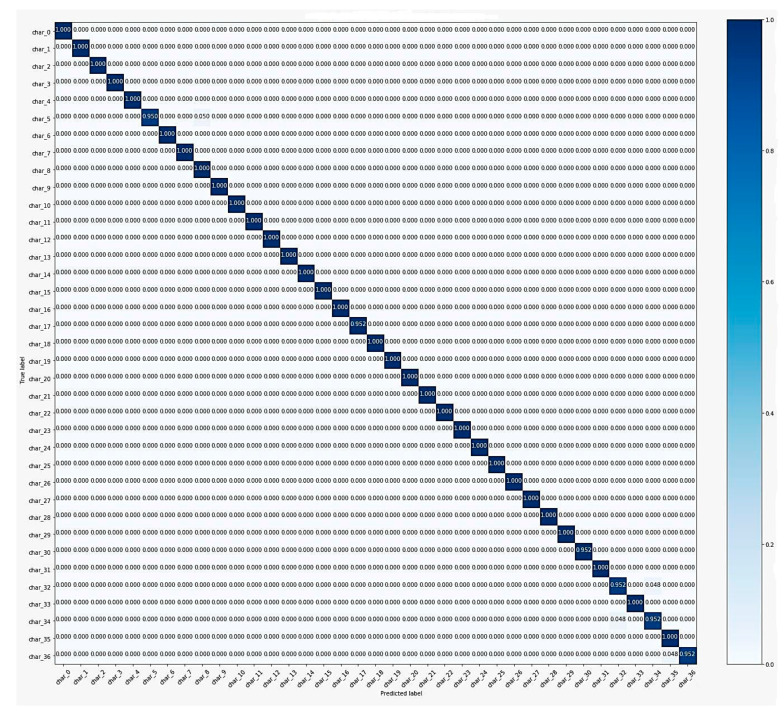
The confusion matrix for Devanagarithat contains 36 classes of characters [54].

Table 5 shows the different datasets fed to our network; we achieved higher accuracy than that of previous studies. It should be noted that we used MNIST and LeNet-5 [46], which is a numeral dataset, however, our model achieved higher accuracy compared to the original implementation. Figure A2 and Figure A3 in the Appendix A show confusion matrices for the Urdu and Devanagari datasets. Our model performs better than the previous implementations. Further more there were additional experimentation were performed that are provided in Table A2 in the Appendix A.

Table 6 summarizes a side-by-side comparison of the most competitive (error rate < 1%) results found in the state of the art for the MNIST database without data augmentation, including our proposed model results. Table 7 shows the details of the number of parameters of these models.

We also calculated the inference time per image for the benchmark models. However, the inference time depends on the architecture of the model, GPU, batch size, and Cudnn library. We used an Nvidia GX1080Ti, a batch size of 32, and Cudnn 7.1 during experimentation. Table 8 shows the average inference time per image of different models on Poha. ResNet 34 took more inference time per image compared to other models due to its deep architecture. The results of off-the-shelf ResNet 18 and 34 were less accurate, but this could easily change if systematic parameter tuning and data enhancements were applied. Our proposed model required the least time among other deep benchmark models. This is because our model has the least layers and is explicitly developed for handwritten character recognition.

Table 9 shows the significance of our model compared to that of other models and approaches. Various approaches are mentioned regarding PHCR, however, our approach achieved high test accuracy.

**Figure 6:**
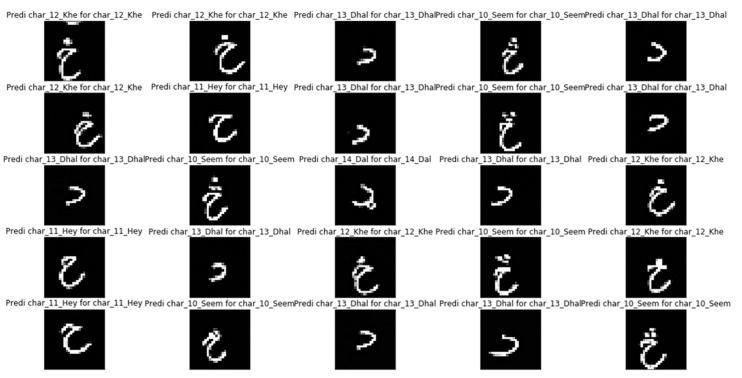
Correct classification of Poha dataset characters by the proposed model.

Figure 6 depicts the correct classification and prediction of Pashto Net for the Poha dataset. The prediction of “Khe”, that is tagged as 12 in sequence is being succfully classified.

**Figure 7:**
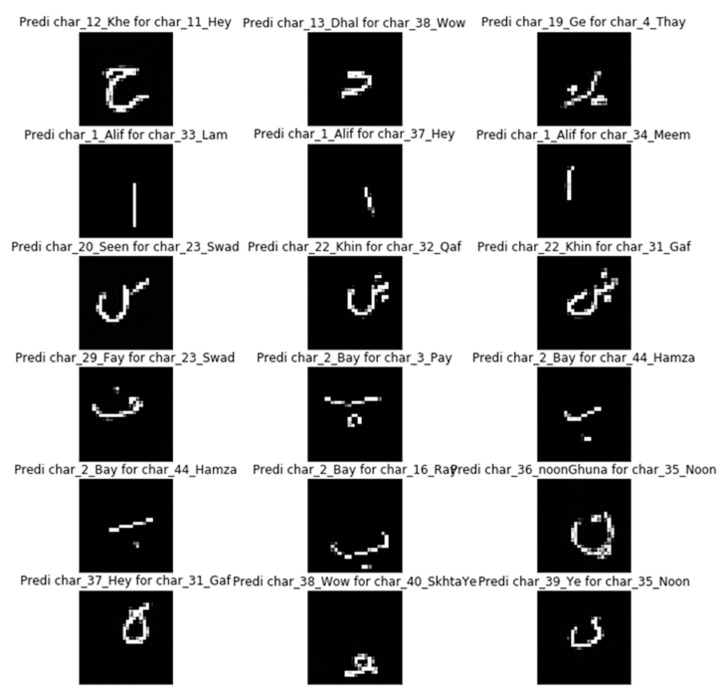
Observed errors in recognition of the Poha dataset by the proposed model.

Figure 7 shows the top 18 incorrect predictions. The errors were caused by the similar cursive morphology of a particular character. For example, in the middle column, the fourth character, which is “Bay”, was predicted as “Pay” due to a close character structure. The difference is the number of dots beneath the horizontal line of a character: the number of dots in “Bay” is one, whereas it is three for “Pay”. It can be seen that the writer drew the dot similar to an open circle, which looks like three small dots. Thus, it is evident that the model performs better in the classification of the Poha dataset characters; however, these mistakes can also be made by the human brain when recognizing and classifying handwritten characters.

The proposed model is significant because it has fewer network parameters. Its testing accuracy is higher than that of many of complex deep models. It is also relatively simple and light-weight, meaning that it ultimately requires fewer computing resources and the execution time is less than that of deep models.

## 5. Conclusions

In this paper, we suggested an optimized CNN for the first time to recognize and classify Pashto handwritten characters. A novel Poha dataset of Pashto handwritten characters was generated. The dataset was further augmented, resulting in significant accuracy during the classification phase. During the experimentation on our suggested dataset using the CNN, the results were compared with different approaches, which not only included Pashto handwritten character, but also Urdu handwritten characters, because these two languages have a close affinity. Pashto handwritten character classification is the first step in the development and design of a learning platform for children and non-native speakers to accurately learn the basics of this language. Moreover, the literature shows a lack of standard datasets for Pashto handwritten characters for comparison and the production of benchmark results. The dataset compiled in the current study was made public so that it can be used by the research community.

The developed CNN resulted in a comprehensive advancement and revolutionary outcomes compared to conventional deep learning approaches. Despite its successful results, some outstanding issues remain; for example, there remains a lack of insight into the specification of the number of hidden neurons and the levels of layers. The requirement of extensive data for a deep network model for validation and efficiency checking is also a potential issue. Therefore, we were required to train our model with extensive data with and without augmented data samples. We suggest that devising optimal network parameters that produce accurate outcomes is also an outstanding research problem. Classification by the suggested model resulted in a number of complex character recognition problems, such as character rotation and noisy images caused by extracting novel features. The classifier can also be assessed using bidirectional LSTM convolutional neural networks. Moreover, we plan to replace our dataset with data generated from generative adversarial networks (GANs) due to the capability of these networks to generate fake data on a large scale. Because GANs and other data science approaches consistently generate large datasets, it is essential to design the CNN model more efficiently to reduce memory usage and enhance resource utilization and computation.

Our suggested model was more efficient than the approaches used in the existing literature in terms of the inference time per image and the number of fine-tuned parameters (Table 7 and Table 8). Furthermore, our suggested model performed efficiently and effectively in character classification and recognition, as evidenced by better accuracy in minimal time compared to previously studied approaches. We strongly suggest the proposed model is suitable for the future development of automated intelligent Pashto handwritten recognition systems, in addition to educational mobile and web applications for children and non-native Pashto speakers.

## Figures and Tables

**Table 1:** Detailed comparison of Urdu handwritten isolated character and word recognition techniques.

Reference	Approach	Features	Classification	Dataset
Pathan et al. [36]	Isolated character	Position of secondary ligatures, and moment, invariant curvature, slope	SVM	36,800 characters
Ali et al. [37]	Words	Stroke variance	Neural Network	25 images
Mukhtar et al. [38]	Ligature	Structural	SVM	1600 words
Sagheer et al. [39]	Numeral	Gradient	SVM	60,329
Basu et al. [40]	Numeral	QTLR	SVM	3,000

**Table 2:** The architecture of the proposed convultional neural network (CNN) model. The table presents the classification report of the Poha dataset on the proposed CNN.

Layers (type)	Output Shape		Parameters		Number of Parameters
Input	28,28,1		-		0
1 (Conv2D)	26, 26, 32		3 × 3 Conv, 32 ReLU		320
2 (MaxPooling)	13, 13, 32		2 × 2 max-pooling, stride 2		0
3 (Conv2D)	11, 11, 64		3 × 3 Conv, 64 ReLU		18,496
4 (MaxPooling)	5, 5, 64		2 × 2 max-pooling, stride 2		0
5 (Conv2D)	3, 3, 64		3 × 3 Conv, 64 ReLU		36,928
6 Flatten	576		-		0
7 Dense	64		ReLU		36,928
8 Dense	44		Softmax		2860
Total parameter: 95,532		Trainable parameters: 95,532		Non-trainable parameters: 0	

**Table 3:** Details of recall, precision, and F1 score on benchmark models with Poha.

Model	Accuracy (%)	Val loss	Recall	Precision	F1 Score
ResNet18	98.21	0.0314	0.98288	0.9901	0.9829
ResNet34	97.48	0.05072	0.9792	0.9893	0.97963
**Proposed**	**99.64**	**0.00981**	**0.9964**	**0.9962**	**0.9964**

**Table 4:** Detailed comparison of Pashto handwritten recognition techniques.

Reference	Approach	Features	Accuracy (%)
[11]	K-Nearest Neighbor	Zoning features	70.05%
[53]	Neural Network	Geometrical strokes	80%
[43]	KNN	Statistical geometrical	74.8%
Proposed approach	CNN	Pixel and geometrical-based	99.64%

**Table 5:** Details of recall, precision, and F1 score on benchmark datasets by the proposed model.

Dataset/Model	Accuracy (%)	Recall	Precision	F1 Score	Pre-Trained Accuracy (%)
Urdu [44]	99.41	0.9942	0.9943	0.99423	96.04
Devanagari [55]	99.53	0.9952	0.9953	0.99523	99.1
MNIST [46]	99.35	0.9931	0.9934	0.9931	95.0
Poha (Proposed Model)	99.6	0.9964	0.9962	0.9964	-

**Table 6:** The comparison of error rates of the most competitive (error rate < 1%) models for the MNIST dataset without data augmentation or preprocessing.

Technique	Test Error Rate
HOPE + DNN with unsupervised learning features [56]	0.40%
Deep convexNet [57]	0.83%
CDBN [58]	0.82 %
S-SC + linear SVM [59]	0.84%
2-layer MP-DBM [60]	0.88%
DNet-kNN [61]	0.94%
2-layer Boltzmann machine [62]	0.95%
Batch-normalized maxout network-in-network [63]	0.24%
Committees of evolved CNN(CEA-CNN) [64]	0.24%
Genetically evolved committee CNNs [65]	0.25%
Committees of 7 neuro-evolved CNNs [65]	0.28%
CNN with gated pooling function [66]	0.29%
Inception-Recurrent CNN + LSUV + EVE [67]	0.29%
Recurrent CNN [68]	0.31%
CNN with piecewise linear activation units [69]	0.31%
CNN (5 conv,3 dense) with full training [70]	0.32%
Simple Conv [71]	0.40%
**Proposed (3 conv, 2 dense) with 50 epochs**	**0.09%**

**Table 7:** Comparison of the total number of parameters.

Model	Number of Parameters	Trainable Parameter	Non-Trainable Parameters
ResNet18	11,186,889	11,178,947	7942
ResnetNet34	3,273,964	21,302,473	15,366
Proposed Approach	95,532	95,532	-

**Table 8:** Comparison of inference time per image using a GTX 1080Ti for benchmark models on the Poha dataset.

Model	Time (ms)
ResNet 34	51.34
ResNet 18	31.72
Proposed Approach	26.56

**Table 9:** Detailed comparison of our approach to Pashto handwritten character classification with other techniques.

Reference	Approach	Features	Accuracy (%)
[11]	K-Nearest Neighbor	Zoning features	70.05%
[43]	KNN	Statistical geometrical	74.8%
[72]	Neural Network	Geometrical strokes	75%
[73]	BPNN, PNN	Geometrical strokes	67%
[73]	BLSTM	Pixel-based	94%-
[42]	CNN	Pixel and geometrical based	96.04%
**Proposed Approach**	**CNN**	**Pixel and geometrical based**	**99.64%**

## Appendix A

**Table A1:** Class wise classification report of the Poha dataset on the proposed model.

Class	Precision	Recall	F1-Score
0	1	1	1
1	1	1	1
2	1	1	1
3	1	1	1
4	1	1	1
5	1	1	1
6	1	1	1
7	1	1	1
8	1	1	1
9	1	1	1
10	1	0.99	0.99
11	1	1	1
12	1	1	1
13	1	0.99	1
14	0.99	1	1
15	1	1	1
16	1	1	1
17	1	1	1
18	1	1	1
19	1	1	1
20	1	1	1
21	0.99	0.98	0.99
22	1	1	1
23	0.99	1	0.99
24	1	1	1
25	1	1	1
26	1	1	1
27	0.99	1	1
28	1	1	1
29	1	1	1
30	1	1	1
31	1	0.99	0.99
32	1	0.99	0.99
33	0.98	1	0.99
34	1	0.98	0.99
35	0.99	1	0.99
36	1	1	1
37	0.99	1	1
38	0.99	0.99	0.99
39	1	1	1
40	0.99	1	1
41	1	1	1
42	1	1	1
43	1	1	1
Avg	0.9964	0.9962	0.9964

Additional experiments:

**Table A2:** Comparison with benchmark deep learning models on the Poha dataset.

Approach	Test Loss	Accuracy (%)
Inception V-3	0.395%	94.32%
Mobile Net	0.070%	98.17%
Desne Net 201	0.0300	99.33%
ResNet50	0.340%	90.53%
**Our Approach**	0.00981%	99.64%
